# Supplementary material for: Two Be or Not Two Be: The Nuclear Autoantigen La/SS-B Is Able to Form Dimers and Oligomers in a Redox Dependent Manner
Source: Int J Mol Sci. 2021 Mar 25;22(7):3377. doi: 10.3390/ijms22073377 (PMC8036718; doi:10.3390/ijms22073377)
Supplement: Supplementary file 1 [file ijms-22-03377-s001.pdf]

Supplemental Figures

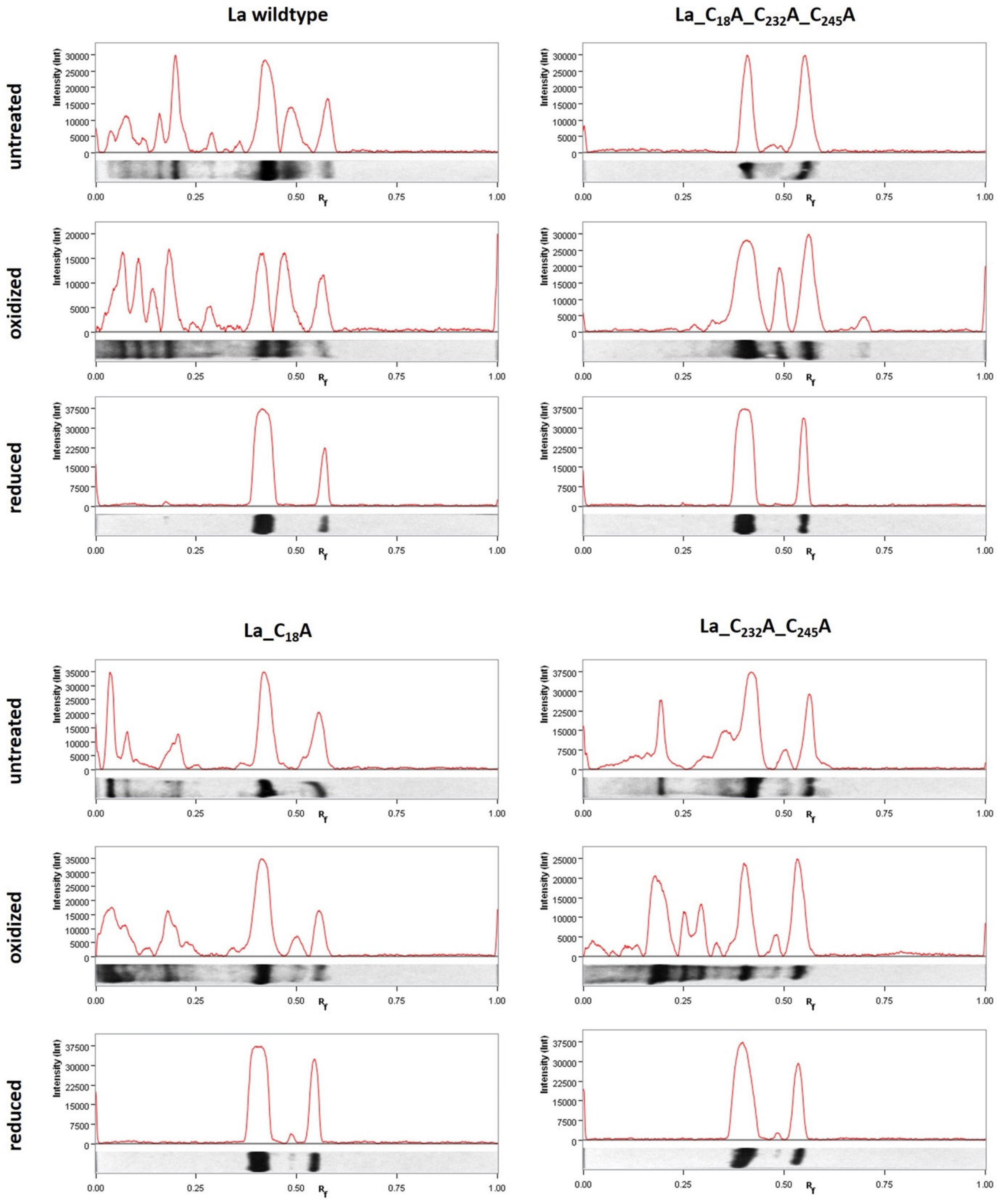

Supplemental Figure 1(A). Densitometric evaluation of the immunoblot shown in Figure 7 (C) obtained with the anti-La mAb SW5.

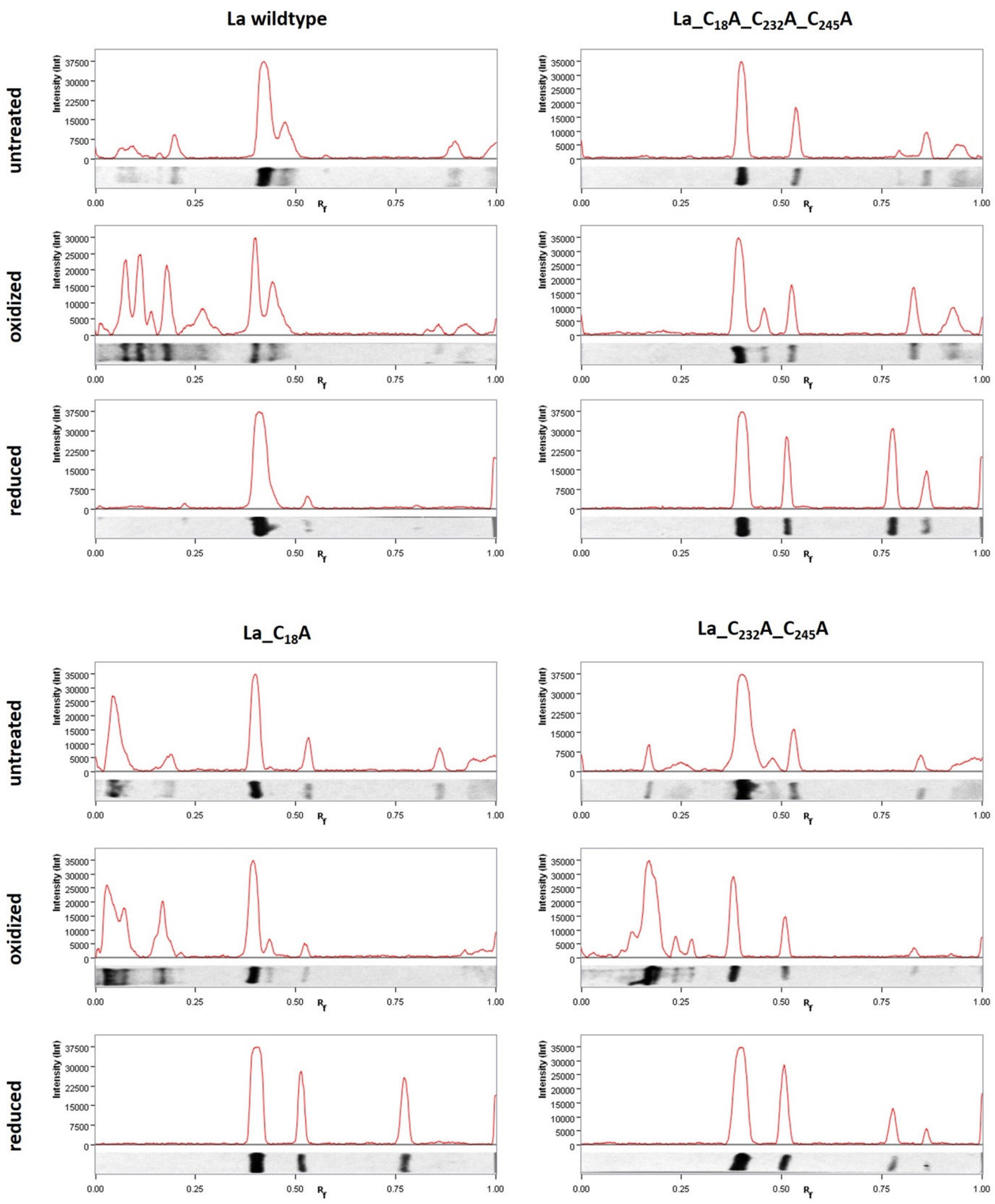

Supplemental Figure 1(B). Densitometric evaluation of the immunoblot shown in Figure 7 (C) obtained with the anti-La mAb 7B6.
